# Correlation of serum interleukin-8 with disease severity in aplastic anemia

**DOI:** 10.1016/j.htct.2025.103989

**Published:** 2025-10-08

**Authors:** Shahla’a Fadhil Sabir, Huda Ibraheem Abd AL-Lateef, Naam Ali Hamza

**Affiliations:** Mustansiriyah University, The National Center of Hematology, Baghdad, Iraq

**Keywords:** Aplastic anemia, Severity of disease, Chemokine, Interleukin-8

## Abstract

**Background:**

Aplastic anemia is a heterogeneous group of hematological disorders identified by the presence of one or more cytopenias in the bone marrow or the peripheral blood or both, affecting either one or multiple blood cell lineages. The pathophysiology of this disorder is unclear; however, the prevailing hypothesis posits that an aberrant immune response is responsible for the death of hematopoietic precursor stem cells due to autoreactive cytotoxic T lymphocytes in individuals with a genetic predisposition. Interleukin-8 acts as a pleiotropic prototype chemokine and serves as a powerful inhibitor of myelopoiesis and assumes an essential role in both the initiation and progression of acute inflammation and tissue damage. Consequently, it is postulated that the sustained elevation in interleukin-8 production could potentially lead to immune-mediated bone marrow failure in aplastic anemia. The aim of this study was to evaluate the serum interleukin-8 concentrations in individuals with aplastic anemia as well as investigate its potential connection with disease severity.

**Methods:**

This study was performed at the National Center of Hematology and included 28 aplastic anemia patients and 30 healthy individuals matched by age and gender as a control group. The serum interleukin-8 levels were measured by the quantitative sandwich enzyme-linked immunosorbent assay (ELISA) method.

**Results:**

Considerrable elevation in serum interleukin-8 levels were observed between the two groups (p-value = 0.405). Of the aplastic anemia patients, severe cases had significantly higher levels of interleukin-8 compared to non-severe patients (p-value = 0.0495), with a cutoff serum level of 7.71 pg/mL.

**Conclusion:**

Interleukin-8 may have a role in the immune-mediated pathophysiology of aplastic anemia as well as have a significant correlation with disease severity.

## Introduction

Idiopathic acquired aplastic anemia (AA), an uncommon and potentially fatal bone marrow failure, is identified by hypocellular bone marrow and peripheral blood cytopenias [1]. Its effects may range in severity from mild to severe cytopenia [2]. AA is classified into three clinical and hematological categories, which are determined by evaluating the cellularity of the bone marrow (BM), platelet count, absolute neutrophil count (ANC), and reticulocyte count [3]. AA is a disease based on an immune meditated pathway in which the dysfunctional effector cells recognize and destroy the marrow progenitor cells, resulting in pancytopenia [4,5]. The immunopathology theory of AA is supported by the positive response to immunosuppressive therapy with the administration of anti-thymocyte globulin and cyclosporine [5]. A variety of growth factors and cytokines, specifically chemokines, synthesized by stem cells, regulatory cells, and marrow stromal cells, play vital roles in the BM microenvironment. These components exert regulatory control over hematopoiesis through autocrine and paracrine mechanisms [6,7]. Interleukin-8 (IL-8), a pleiotropic prototype CXC chemokine, is produced by different cell types, primarily by macrophages and fibroblasts in the BM, and neutrophils and T-cells in the peripheral blood [8,9]. Via a receptor-ligand mediated mechanism, IL-8 may contribute to the suppression of hematopoiesis, a process by which the blood cells are originated [8]. It serves as a powerful inhibitor of myelopoiesis and assumes an essential role in both the initiation and progression of acute inflammation and tissue damage [10,11]. Research conducted on the pathophysiology of the disease has predominantly centered on stem cell abnormalities and immunological pathways, while, limited data exists regarding the involvement of the BM microenvironment in this condition [1]. Consequently, it is postulated that the sustained elevation in IL-8 production could potentially play a role in the onset of immune-mediated BM failure in individuals with AA [11]. Furthermore, evidence shows that IL-8 correlates with disease severity and progression [12]. This study evaluates the serum IL-8 concentrations in individuals with AA as well as investigates potential connections with disease severity.

### Patients and methods

The present study was carried out from December 2020 to December 2022 at the National Center of Hematology, and included 28 patients with newly diagnosed idiopathic AA. Their initial diagnostic data was established from their medical records at the time of sampling. Another 30 apparently healthy individuals, with similar ages and genders, were chosen as a control group. The researchers received ethics approval and only subjects who gave written consent were included in the study. Patient data comprised an analysis of blood components, including a complete blood count, assessment of peripheral blood smears, and testing of BM samples. Other criteria for the inclusion of patients were: no organomegaly, normal renal function, normal liver function, normal Erythrocyte Sedimentation Rate (ESR), a negative serological connective tissue screen, no history of acute febrile illness, and no evidence of organ dysfunction. AA patients were categorized into mild, moderate, severe and very severe. Severe AA was characterized by a reduction in blood counts affecting two or more hematopoietic lineages (i.e., absolute reticulocyte count <60 × 10^9^/L, an ANC <0.5 × 10^9^/L, or platelet count <20 × 10^9^/L) that is accompanied by bone marrow hypocellularity, defined as less than 25 % of the normal cellularity. Very severe AA was identified by an ANC <0.2 × 10^9^/L. While, the decrease in blood counts that did not meet the criteria for severe disease was classified as moderate AA [13]. Both patients and controls were free from chronic illness like diabetes, hypertension and recent febrile illness, as well as serological evidence of acute or chronic viral hepatitis.

Under aseptic circumstances, 3 mL of peripheral blood were drawn from both patients and controls, then dispensed in sterile plain tubes, and centrifuged for ten minutes at 4000 rpm. The serum samples were collected and stored at −20 °C until the IL-8 level was analyzed. As per the instructions provided by the manufacturer, the levels of IL-8 for all participants were measured using a commercial quantitative sandwich ELISA assay kit (R&D Systems, USA). The concentration in pg/mL was used to express the results.

### Statistical analysis

The Statistical Analysis System (SAS 2012) program was employed to detect the effect of differences between the groups. The Mann-Whitney test was employed for non-normally distributed continuous variables and a *t*-test was employed for normally distributed continuous variables. Correlation coefficients between variables were estimated in this study. A p-value ≤0.05 was considered statistically significant, whereas a p-value ≤0.01 was considered highly significant [14].

## Results

Twenty-eight AA patients were included in this study with a median age of 36.5 ranging from 8–68 years. Of these patients, 15 (53.6 %) were male and 13 (46.4 %) were female with a M:F ratio of 1:1. Thirty samples of apparently healthy volunteers were included as a control group in the analysis with median age of 36.5 ranging between 17 and 67 years and a M:F ratio of 1:1 (Table 1).

**Table 1:** General characteristics of aplastic anemia patients and the control group.

	Patient(*n* = 28)	Control(*n* = 30)	p-value
Age in years - median (Range)	36.5 (8–68)	36.5 (17–67)	0.8260*
Sex - n ( %)			0.7993**
Male	15 (53.6 %)	15 (50 %)	
Female	13 (46.4 %)	15 (50 %)	
Hemoglobin (g/dL) Mean ± SE (Range)	7.225 ± 0.2803 (5.1–10)	–	
Platelet x 10³/µL Mean ± SE (Range)	20.79 ± 1.816 (7-44)	–	
ANC x 10³/µL Mean ± SE (Range)	583.9 ± 53.68 (110–1100)	–	

SE: Standard error; ANC: absolute neutrophil count.

⁎ *t*-test.

⁎⁎ Fisher's exact test.

**Table 2:** Comparison of mean interleukin-8 (IL-8) serum level between aplastic anemia patients and controls.

Group	Mean ± SE IL-8 (pg\mL)
Aplastic Anemia (*n* = 28)	15.02 ± 2.81
Control (*n* = 30)	10.19 ± 1.01
p-value	0.405

Mann-Whitney test was used for statistical analysis. Test is considered significant if p-value ≤0.05.

**Table 3:** Relationship between severity of disease and mean serum level of interleukin-8 (IL-8) in aplastic anemia patients.

Severity of disease	Mean ± SE IL-8 (pg\mL)
Severe (No = 21)	17.54 ± 3.59
Non-severe (No = 7)	7.43 ± 0.52
p-value	0.0149

Mann-Whitney test was used for statistical analysis. Test is considered significant if p-value ≤0.05.

Regarding the IL-8 mean serum level, there was an increase in the serum level of IL-8 in AA patients in comparison to the control group (p-value = 0.405). However, data indicate a significant difference in mean IL-8 (p-value = 0.0149) between severe and non-severe AA patients (Tables 2 & Table 3).

Moreover, a significant difference in mean serum level of IL-8 was observed between different age groups of patients with AA; higher mean serum levels were found in older patients (p-value = 0.0406). While there was no statistically difference observed in the mean serum level of IL-8 between sexes in AA patients (Tables 4 & 5).

**Table 4:** Relationship between gender and mean serum level of interleukin-8 (IL-8) of aplastic anemia patients.

Sex	Mean ± SE IL-8 (pg\mL)
Male	15.76 ± 3.93
Female	14.14 ± 4.16
p-value	0.383

T-test was used for statistical analysis. Test is considered significant if P-VALUE ≤0.05.

**Table 5:** Relationship between age groups and mean serum level of interleukin-8 (IL-8) of aplastic anemia patients.

Age (Year)	Mean ± SE IL-8 (pg/mL)
≤40	8.31 ± 0.4492
>40	22.25 ± 5.922
p-value	0.0406

T-test was used for statistical analysis. Test is considered significant if p-value ≤0.05.

A non-statistically significant (p-value >0.05) correlation coefficient was observed between IL-8 levels and other patient parameters, such as hemoglobin, platelet count, and ANC (Table 6).

**Table 6:** Correlation coefficient between interleukin-8 (IL-8) and different hematological parameters among aplastic anemia patients.

Parameters	Correlation coefficient (r) with IL-8	p-value
Hemoglobin	−0.18	>0.05
platelet count	−0.04	>0.05
ANC	−0.03	>0.05

Receiver operating characteristic (ROC) analysis showed that IL-8 with a cutoff value of 7.71 pg/mL was highly significant with good sensitivity and specificity to discriminate between severe and non-severe AA patients (Table 7).

**Table 7:** Receiver operating characteristic (ROC) analysis evaluating the diagnostic accuracy of interleukin-8 levels in aplastic anemia patients.

Interleukin-8	AUC	Explanation	AUC at 95 % CI	p-value	Optimum cut-off value	SP ( %)	SN ( %)
Aplastic Anemia versus Control	0.564	Poor	0.415–0.714	0.401	8.33 pg/mL	50	64.3
Severe versus non-severe	0.806	Good	0.637–0.975	0.017	7.71 pg/mL	71.4	81

AUC: Area under curve; 95 % CI: 95 %confidence interval; SN: sensitivity; SP: specificity.

## Discussion

IL-8 has the ability to suppress the production of myeloid progenitor cells by impeding their proliferation through a mechanism involving a receptor-ligand interaction (transmitting an inhibitory signal), specifically, IL-8 (CXCR-2), which is expressed on progenitor cells [15]. Since IL-8 has strong stimulatory effects on neutrophils [16], elevated concentrations of this cytokine in the blood stream have the potential to promote activation-induced apoptosis of fully developed neutrophils in the peripheral system [17].

This study shows that AA patients have elevated, albeit non-significant, mean serum IL-8 concentrations in comparison to the control group (p-value >0.05). Previous studies conducted in pediatric and adult patients by Gupta et al., Bhargawa et al., and Singh et al. revealed significant increments (p-value <0.001) in the IL-8 level of AA patients in comparison to controls (15.58 ± 18.0 versus 1.85 ± 0.95, 122.56 ± 97.79 versus 3.42 ± 1.73, and 120.28 ± 94.98 versus 1.79 ± 0.78, respectively). A recent study confirms these results showing that the median IL-8 level is significantly higher in AA patients than in a control group (320 versus 97; p-value <0.0001) [2,12,18,19]. These findings may give us an idea about the pathology of the AA and the role of cytokines like IL-8 in disease activity and marrow suppression.

Additionally, in the current study, patients diagnosed with AA showed significantly increased mean serum levels of IL-8 in severe AA patients, signifying that IL-8 levels may be playing a role in exacerbating disease progression and severity. This finding agrees with many other studies such as those by Gupta et al., Bhargawa et al., Singh et al., and Khalifa et al., that revealed a significant correlation between an elevated level of IL-8 and the severity of the disease [2,12,18,19]. A study conducted by Tripathy et al. examining bone marrow and peripheral blood samples obtained from AA patients, demonstrated an increase of IL-8 in both samples that correlated with disease severity [8]. Tang et al. also demonstrated the same result using peripheral blood samples [20].

There are several studies that revealed a substantial association between elevated levels of IL-8 in peripheral blood and the severity of the disease. Therefore, IL-8 could have a significant role in the pathogenesis of a severe type of idiopathic AA [18,21]. The present study confirmed other studies suggesting that patients with AA may have an inflammatory background mediated by IL-8 acting as a negative regulator of hematopoiesis [19]. Further studies are encouraged to explore a targeted therapy that inhibits IL-8 in the context of hematological and immunological settings.

Higher levels of IL-8 were detected in the older age group in comparison to patients aged between 20–40 years. This finding is inconsistent with the data on serum IL-8 levels detected in children diagnosed with AA, as elevated concentrations were associated with younger severe AA patients [18].

There was no statistically significant relationship between the mean IL-8 serum level and the mean levels of hemoglobin, platelets, and ANC in the AA patient group. The relationship between IL-8 and peripheral blood indices might not be consistent across patients due to heterogeneity in disease pathophysiology or the severity of AA.

When combining other investigations with our findings, it is evident that patients diagnosed with severe and very severe AA exhibit considerably higher peripheral blood IL-8 levels compared to those with non-severe AA. Moreover, these elevated IL-8 levels demonstrate a positive correlation with disease severity, suggesting a crucial role in the pathogenesis of AA [9,12,19]. Thus, IL-8 might be useful as a discrimination marker with the current study suggesting that the cutoff value of 7.71 pg/mL may separate severe from non-severe AA.

One of the primary limitations of this study is the limited sample size only from a single institution. Consequently, further research involving a large sample size from many institutions is imperative to enhance the generalizability of these findings. Additionally, the lack of patient follow-up after therapy in this study represents a constraint that should be acknowledged.

## Conclusion

IL-8 may have a role in the clinical presentation and severity of the AA; the immune-mediated action of IL-8 in patients diagnosed with AA may be associated with an increment in BM hypocellularity and subsequently more profound peripheral blood cytopenias.

## Data availability

The datasets generated during and/or analyzed during the current study are available from the corresponding author on reasonable request.

## Funding

Self-funded.

## Consent

Informed consent was obtained from each participant prior to enrollment in this study

## Final approval of manuscript

All the authors.

## Ethics approval

Ethics approval is sought from the scientific ethical committee in the National Center of Hematology/ Mustansiriyah University (Reference: nch-erc-20-27), and it conformed to the ethics guidelines of the Declaration of Helsinki.

## CRediT authorship contribution statement

**Shahla’a Fadhil Sabir:** Methodology, Data curation, Project administration, Writing – review & editing. **Huda Ibraheem Abd AL-Lateef:** Project administration, Writing – review & editing. **Naam Ali Hamza:** Project administration, Writing – original draft.

## Conflicts of interest

The authors declare no competing interests.
